# Spectrokinetic characterization of photoactive yellow protein films for integrated optical applications

**DOI:** 10.1007/s00249-019-01353-8

**Published:** 2019-03-23

**Authors:** Szilvia Krekic, Dávid Nagy, Stefka G. Taneva, László Fábián, László Zimányi, András Dér

**Affiliations:** 1 Institute of Biophysics, Biological Research Centre, Hungarian Academy of Sciences, Temesvári krt. 62, P.O. Box 521, Szeged, 6701 Hungary; 2 Institute of Biophysics and Biomedical Engineering, Bulgarian Academy of Sciences, Acad. G. Bonchev Str., bl. 21, Sofia, 1113 Bulgaria

**Keywords:** Photoactive yellow protein, Integrated optics, Biophotonics, Kinetic absorption spectroscopy, Optical waveguide lightmode spectroscopy

## Abstract

In this paper, the photocycle of the dried photoactive yellow protein film has been investigated in different humidity environments, in order to characterize its nonlinear optical properties for possible integrated optical applications. The light-induced spectral changes of the protein films were monitored by an optical multichannel analyser set-up, while the accompanying refractive index changes were measured with the optical waveguide lightmode spectroscopy method. To determine the number and kinetics of spectral intermediates in the photocycle, the absorption kinetic data were analysed by singular value decomposition and multiexponential fitting methods, whose results were used in a subsequent step of fitting a photocycle model to the data. The absorption signals of the films were found to be in strong correlation with the measured light-induced refractive index changes, whose size and kinetics imply that photoactive yellow protein may be a good alternative for utilization as an active nonlinear optical material in future integrated optical applications.

## Introduction

In the past few decades, there has been an ever increasing demand for faster information transmission and data processing. Achieving greater speed in state-of-the-art electronic devices requires further miniaturization of integrated electronic circuits and integrating more and more components on a small silicone chip. However, scaling down the structure causes several problems (heat damage, crosstalk, quantum effects, etc.), limiting the size of the components. The empirical law proposed by Moore (1965), stating that the number of electronic components integrated on a silicon wafer of unit area is doubling in every 18 months, has been valid until recent times, but cannot be sustained for much longer (see Waldrop 2016 as a quick overview).

One of the several alternative solutions investigated is integrated optics (IO), which comprises similar elements to integrated electronics; however, in IO the information transmission and processing is done solely by optical means. Analogously to their electronic counterparts, IO circuits also comprise passive and active elements, corresponding to the wires, resistors and capacitors, on the one hand, and the transistors, on the other. The theory of integrated optics and the technology for manufacturing passive IO elements (i.e. miniature waveguides) are well established, and the main challenge is to find or develop materials with suitable nonlinear optical (NLO) properties that can function as active elements in IO circuits.

In recent works, cost-effective organic materials have been proposed to be tested as active IO components, as alternatives to the currently used semiconductor materials (Haque and Nelson 2010). The design and application of organic, pi-conjugated molecular materials generated the most interest (Hales et al. 2010; Hu et al. 2008; Service 1995), but their naturally occurring, stable counterparts, such as chromoproteins, have also been successfully tested (as a recent review, see Fábián et al. (2015)). One of the most researched biological materials in the field is the light-sensitive protein bacteriorhodopsin (bR) (Oesterhelt and Stoeckenius 1971, 1973), due to its relatively large light-induced refractive index change (Ormos et al. 2002) and mechanical stability (Hristova et al. 1984). In previous works of our group, the usability of bR in IO circuits has been investigated extensively, demonstrating the ability of fast optical switching and light modulation (Dér et al. 2007; Fábián et al. 2010, 2011; Mathesz et al. 2013). In our current work, similarly to bR, the expedience of the photoactive yellow protein (PYP) in IO circuits has been investigated.

PYP is a small, water-soluble cytoplasmic protein that was first extracted from the purple halophilic bacterium *Halorhodospira halophila* (Meyer 1985; Meyer et al. 1987). The chromophore of the PYP is *p*-coumaric acid, which is responsible for the colour of the protein that, when excited by light, undergoes a reversible trans–cis isomerization around its thiol-ester linked bond attached to cysteine-69 (Baca et al. 1994; Hoff et al. 1994a; Van Beeumen et al. 1993). The photoexcitation is also linked to the negative phototaxis detected in bacteria where the PYP is present as photoreceptor (Sprenger et al. 1993). Upon photoisomerization PYP undergoes a cyclic reaction series between quasi-stable intermediate states, accompanying transient protonation and conformational changes before returning to its original state (“pG”). The intermediate states of the photocycle may also differ spectrally. The pG initial or dark state has an absorption maximum at 446 nm, while during the photocycle, two spectrally distinct states are present: the red-shifted pR, followed by the blue-shifted pB. Since the first study of the photocycle (Meyer et al. 1987, 1989), the kinetic and spectral properties of the intermediates have been investigated with numerous methods under various environmental conditions, resulting in several distinctive photocycle schemes and rate coefficients, including spectrally “silent” transitions, too (Borucki et al. 2006; Cusanovich and Meyer 2003; Groot et al. 2003; Hellingwerf et al. 2003; Hoff et al. 1994b; Ihee et al. 2005; Imamoto and Kataoka 2007; Joshi et al. 2005; Kim et al. 2012; Pande et al. 2016; Ujj et al. 1998; van der Horst et al. 2005; Yang et al. 2017). A recent work suggests that the protein’s photodynamics also depend on the wavelength of the excitation light (Mix et al. 2018). Taking into account all internal and external factors, the photocycle can occur multiple ways.

When considering PYP for IO applications, the most important features are the fast spectral transitions of the protein at the beginning of its photocycle and the accompanying refractive index changes. Contrary to bR that is available in large membrane fragments, or in a detergent-solubilized form, PYP is water-soluble per se, which offers unique opportunities for its combination with passive IO elements. These properties make PYP a good candidate for using it as an active element of IO structures. For such applications, dry samples are preferred, because of their higher mechanical stability and larger photoinduced refractive index changes.

In our current work, we investigated the photocycle of dry PYP films and determined the accompanying light-induced refractive index changes at various relative humidities in order to evaluate their potential IO adaptability. For the photocycle measurements, we used an optical multichannel analyser (OMA) set-up, while the refractive index change measurements were performed with the optical waveguide lightmode spectroscopy (OWLS) method. Our results show that PYP films of controlled relative humidity are good candidates for utilization as active elements in IO devices.

## Materials and methods

### Sample preparation

PYP was a kind gift of Dr. John Fitch. The lyophilized protein was dissolved in 10 mM Tris buffer, pH 8.2, up to a protein concentration of 50 μM. In both experiments, 15 µl protein solution was homogeneously dried on a glass slab forming an approximately 5 mm diameter patch. For kinetic absorption spectroscopy, the glass slab with the dried protein film was placed into a plastic cuvette. Saturated salt solutions were introduced in the bottom of the cuvette, without contact with the PYP film, to maintain the desired humidity. For the low-relative humidity (20%) experiments, we used potassium acetate, while high-relative humidity (85%) was achieved by using potassium chloride.

For the OWLS experiments, the protein solution was dried on the top of a slab optical waveguide directly over the grating coupler. The waveguide and the rotational table of the experimental set-up were placed inside a glass case, where the environment’s humidity was also controlled with the same salt solutions as used in the absorption kinetics experiments. An additional experiment was performed in an environment where the relative humidity was kept at 75% using sodium chloride.

Samples were prepared 1 day before the experiments to attain the desired humidity and hydration state of the protein.

### Kinetic absorption spectroscopy

Excitation of the protein film was done by a Surelite I Nd-YAG laser with an OPO extension (Continuum, USA). 5-ns pulses, with 5 mJ/cm^2^ at a wavelength of 450 nm, were used, while the white measuring light was provided by a 35-W high-pressure Xenon lamp (Hamamatsu, Japan), which was chopped by a Uniblitz digital shutter to avoid excess exposure of the sample. The shutter was open for 20 ms, and a new recording was done every 6 s to ensure that the protein returned to its dark state between consecutive actinic pulses. The excitation pulse and the measuring light crossed the sample perpendicular to each other. Absorption difference spectra were detected with an Andor iStar gated ICCD detector (Andor Technology, UK) which was attached to a HR-320 spectrograph (ISA Jobin–Yvon, France). The length of the gate pulse of the detector was adjusted depending on its time delay after the laser pulse, and 10–30 spectra were averaged at each delay.

### Data analysis

The data analysis of the OMA experiments was based on the analysis and results in our previous article (Khoroshyy et al. 2013).

The measured time-resolved difference spectra were collected in a $\varvec{D}_{{\varvec{m} \times \varvec{n}}}$ matrix, where *m* is the number of data points on the wavelength scale and *n* is the number of different time delays after the pulsed excitation. Using the Beer–Lambert law, the matrix consisting of the difference spectra can be written as the product of the intermediate difference spectra and the transpose of their time-dependent concentrations.

Singular value decomposition (SVD) was performed on the data matrix to determine the number of spectrally distinguishable intermediates, which is equal to the rank of the matrix, and to reconstruct the data matrix with reduced noise from the significant spectral and kinetic eigenvectors. Assuming first-order transitions between the intermediates, their concentrations are composed of the linear combinations of time-dependent exponentials. As a consequence, the temporal SVD eigenvectors are also linear combinations of the same exponential functions. Multiexponential least squares fit of the temporal eigenvector matrix yielded amplitudes and phenomenological rate coefficients. The data matrix itself was then reconstructed as the linear combination of the obtained time-dependent exponentials.

A spectrotemporal least squares model fit was performed to the matrix $\varvec{D}^{\varvec{e}}$, i.e. the spectra that were reconstructed from the exponential fit of the significant SVD kinetic vectors. The fitting parameters consisted of the spectra of the photocycle intermediates and the rate coefficients of our photocycle scheme obtained by Khoroshyy et al. (2013) for the PYP in solution, and the protein fraction entering into the photocycle. The spectral fitting parameters were permitted to moderately vary relative to the input spectra, with the constraint of no negative absorption, to allow spectral differences between the photocycle intermediates observed in aqueous solution or in the dry, rehydrated sample. As the result of the fit, we could determine the spectra of the different intermediates in the photocycle and the molecular rate coefficients represented in our scheme and also calculate the concentration matrix, i.e. the kinetics of the intermediates. These data were then used in the interpretation of the refractive index changes during the photocycle measured by optical waveguide lightmode spectroscopy.

### Optical waveguide lightmode spectroscopy

To determine the relative refractive index change of the PYP intermediates at different time delays after excitation, we used the optical waveguide lightmode spectroscopy (OWLS) method, which uses the principle that only such discrete modes can propagate in an optical waveguide that meet the criteria of the (1) and (2) mode equations.

1 $$\frac{2\pi }{{\lambda_{0} }}d_{\text{F}} \sqrt {n_{\text{F}}^{2} - N_{\text{TE}}^{2} } = \arctan \sqrt {\frac{{N_{\text{TE}}^{2} - n_{\text{S}}^{2} }}{{n_{\text{F}}^{2} - N_{\text{TE}}^{2} }}} + \arctan \sqrt {\frac{{N_{\text{TE}}^{2} - n_{\text{A}}^{2} }}{{n_{\text{F}}^{2} - N_{\text{TE}}^{2} }}} + m\pi$$

2 $$\frac{2\pi }{{\lambda_{0} }}d_{\text{F}} \sqrt {n_{\text{F}}^{2} - N_{\text{TM}}^{2} } = \arctan \left[ {\frac{{n_{\text{F}}^{2} }}{{n_{\text{S}}^{2} }}\sqrt {\frac{{N_{\text{TM}}^{2} - n_{\text{S}}^{2} }}{{n_{\text{F}}^{2} - N_{\text{TM}}^{2} }}} } \right] + \arctan \left[ {\frac{{n_{\text{F}}^{2} }}{{n_{\text{A}}^{2} }}\sqrt {\frac{{N_{\text{TM}}^{2} - n_{\text{A}}^{2} }}{{n_{\text{F}}^{2} - N_{\text{TM}}^{2} }}} } \right] + m\pi .$$

In the above equations, *λ*_0_ is the vacuum wavelength of the light propagating inside the guiding layer with a refractive index of *n*_F_ and thickness of *d*_F_, while the substrate’s refractive index is *n*_S_. The *m* is the mode order which was zero in all of our experiments. The propagating modes are also dependent on the refractive index of the adlayer (*n*_A_), which is the material that is above the guiding layer. In our experiments, the PYP film was the adlayer. The propagation properties of the guided modes can be represented by the effective refractive indices, which are *N*_TE_ for the transversal electric (s-polarized) and *N*_TM_ for the transversal magnetic (p-polarized) modes.

Our experimental set-up was arranged in a way that the change of the effective refractive index of the guided mode could be easily determined by measuring the incident angle of the light coupled into the waveguide. The arrangement consisted of a slab optical waveguide fixed on a high-resolution (∼ 10^−3^°) rotational table. As a probe we used a HeNe laser (632.8 nm), which was incident on the integrated grating coupler. This wavelength stands outside of the absorption bands of all the intermediate states, so no absorption change interferes with the measurement of the refractive index change. The incoupled light intensity was measured by two photodiodes that were attached to the two ends of the waveguide.

The change in the adlayer’s refractive index alters the coupling conditions in Eqs. (1) and (2). Based on the measured angle shifts of the incoupling peak, the refractive index change could be determined up to a precision of 10^−5^.

We measured the light-induced refractive index change of the dried PYP film with both continuous and pulse excitations. For the CW excitation, a diode laser of 410 nm was used. The pulse excitation was done by a XeCl excimer laser-pumped Coumarin 450 dye laser (wavelengths of 308 and 451 nm, respectively).

## Results and discussion

### Kinetic absorption spectroscopy

The absorption difference spectra were measured in the case of both high humidity and low humidity, but extensive data were only measured when the protein was in a state of high hydration. Our analysis is demonstrated on the high-humidity environment case.

**Fig. 1:**
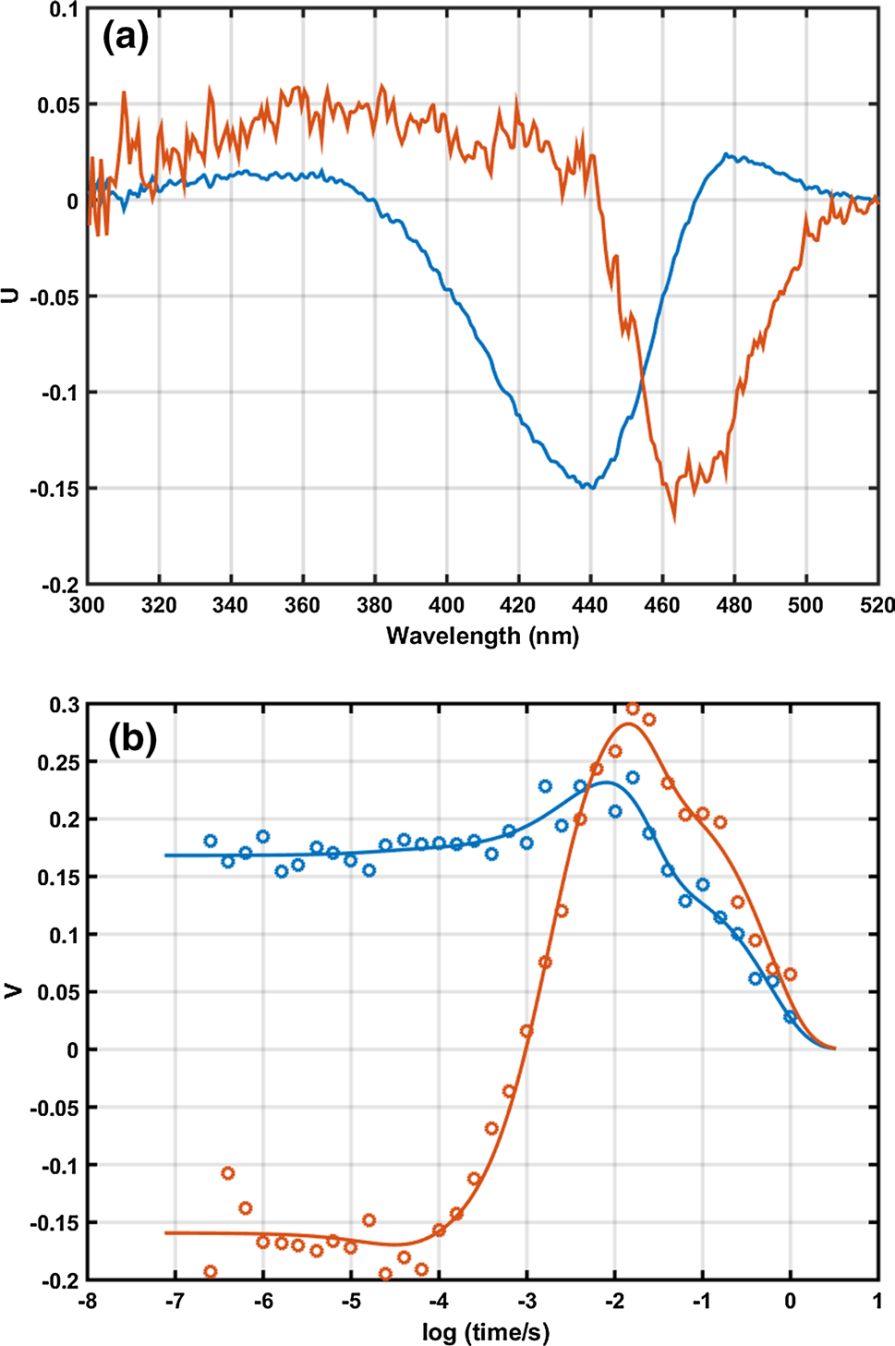
The two significant spectral eigenvectors **a***U*_1_: blue, *U*_2_: red; and the two significant kinetic eigenvectors **b***V*_1_: blue symbols, *V*_2_: red symbols from the SVD analysis. Lines in **b** show the result of the multiexponential fit to the kinetic eigenvectors

Difference spectra were recorded in the range of 250 ns to 1 s in 34 logarithmically equidistant time delays after excitation. SVD yielded a rank of 2, based on the singular values and the autocorrelation of the spectral and kinetic eigenvectors. The first 2 eigenvectors accounted for 96.72% of the variance of the data matrix. Similar experiments on PYP in solution, in the presence or absence of various salts, typically yielded a rank of 3 (Khoroshyy et al. 2013). As it became clear from the analysis, the difference is not due to a reduced number of intermediates, but to the lack of significant spectral difference between certain intermediates in the hydrated PYP film. Global multiexponential fit was done on the two weighted kinetic eigenvectors. For an adequate fit, 5 exponentials were required, similarly to the case of PYP aqueous solutions (Khoroshyy et al. 2013). The significant $\varvec{U}$ spectral eigenvectors and the corresponding $\varvec{V}$ vectors are shown in Fig. 1, together with the multiexponential fit of the latter.

**Fig. 2:**
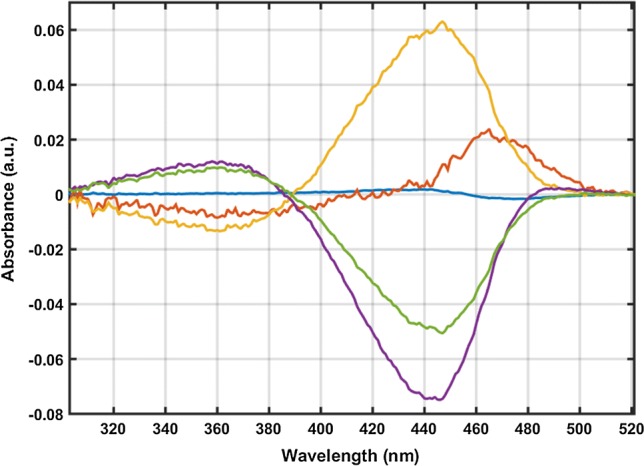
Consecutive exponential amplitude spectra (B-spectra) of the multiexponential fit: blue, first component corresponding to the first (fastest) time-dependent exponential (rate coefficient *k*_1_), red (*k*_2_), yellow (*k*_3_), purple (*k*_4_), green (*k*_5_)

The five exponential amplitude spectra (B-spectra) corresponding to the multiexponential fit (Fig. 2) are by and large similar to those obtained for PYP in solution (cf. Fig. 3A in Khoroshyy et al. 2013). This indicates that not only the number of distinguishable photocycle intermediates (5) but also their spectral characteristics and the photocycle scheme are similar. The corresponding phenomenological rate coefficients are, however, different: $4. 8\cdot 1 0^{ 4}$, $7.0 \cdot 1 0^{ 2}$, $1.44 \cdot 1 0^{ 2}$, 57 and 1.7 s^−1^, as compared to $3.43 \cdot 1 0^{ 5}$, $4. 0\cdot 1 0^{ 3}$, $6. 7 8\cdot 1 0^{2}$, 2.28 and 0.18 s^−1^ in 0.66 M NaCl, pH 8.2, 22 °C. Since the phenomenological rate coefficients are functions of the molecular rate coefficients, the latter are also expected to be different in the dried, hydrated PYP sample compared to the aqueous sample.

**Fig. 3:**
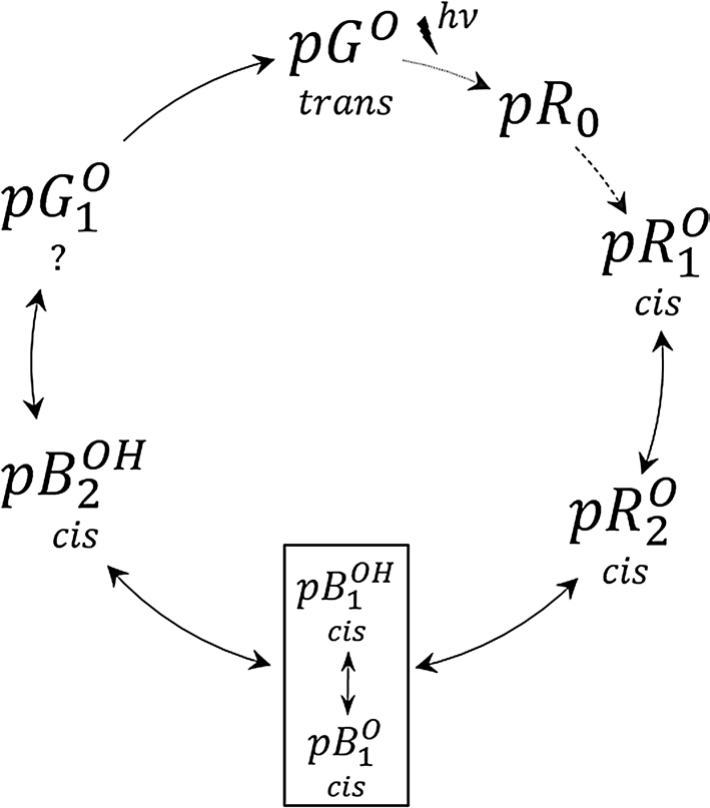
The photocycle scheme. pR and pB intermediates are red- and blue-shifted, respectively, relative to the initial pG states. Trans and cis refer to the isomerization state of the p-coumaric acid chromophore of PYP, and O and OH refer to the deprotonated and protonated states of the chromophore, respectively

Due to the overall similarity of the two data sets, we fitted the photocycle scheme published in Khoroshyy et al. (2013) (Fig. 3) to the present data. As in our earlier publication, pR_O_ was not resolved in this experiment and not included in the photocycle fit.

**Fig. 4:**
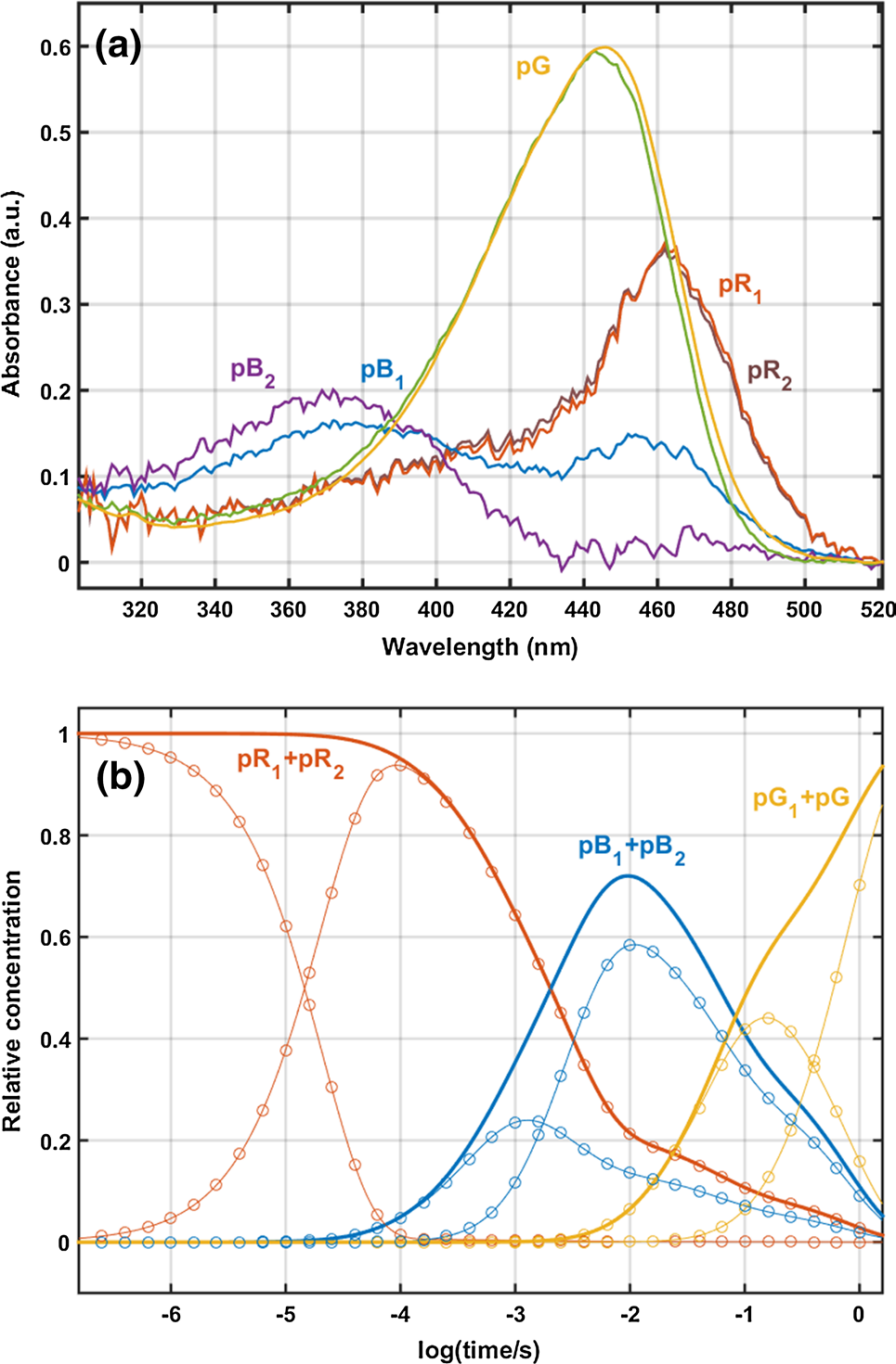
The intermediate spectra obtained from the global spectrotemporal model fit (**a**) and the time-dependent relative concentrations of the intermediates (**b**). In **b,** thin lines show the kinetics of the consecutive individual intermediates and thick lines the total time evolution of the spectrally similar intermediates

The fit yielded the spectra and the kinetics of the photocycle intermediates (Fig. 4a, b) and the molecular rate coefficients of the forward and reverse transitions in the scheme. The obtained intermediate spectra are similar to those published earlier (Khoroshyy et al. 2013, Fig. 4b) with two very similar pR spectra, with pB_1_ appearing as a mixture, indicating a rapid equilibrium between the protonated and deprotonated forms of the chromophore, with a distinct pB_2_ form and a spectrally silent pG_1_ as the final intermediate before the recovery of the dark state, pG. A detailed description of the photocycle intermediates is presented in Khoroshyy et al. (2013). Briefly, the gradual conformational change (opening) of the protein starts already during the pR_1_ to pR_2_ transition, with a very minor spectral change not clearly resolved in this study. The blue shift of the spectrum is known to be due primarily to the protonation of the chromophore. It has also been shown (Khoroshyy et al. 2013) that the main conformational change appears during the pR_2_ to pB_1_ transition. The existence of the pG_1_ intermediate with a spectrum similar to that of pG, the initial form, is supported by the biphasic recovery of the initial form (decay of pB) without a clear spectral signature. The pG_1_ to pG transition is expected to complete the recovery of the initial conformation of the protein without affecting the immediate surroundings of the chromophore. The main difference as compared to the aqueous sample is that pB_2_ is not blue-shifted relative to pB_1_. The approximately 15 nm blue shift in solution has been explained by the completion of the major conformational change in the photocycle, resulting in stronger hydrogen bonding or hydration of the chromophore (Hendriks et al. 2003). This transition has not been observed in PYP crystals (Yeremenko et al. 2006), and it appears to be absent in the dried, partially hydrated PYP film, too. The absence of this conformational change in the present experiment is the probable reason for the faster recovery of the dark state, already reflected in the one order of magnitude faster fifth phenomenological exponential component, 1.7 s^−1^ vs. 0.18 s^−1^. Table 1 lists the molecular rate coefficients obtained for the dried, partially rehydrated sample and the aqueous sample in 0.66 M NaCl, pH 8.2, 22 °C (Khoroshyy et al. 2013). Comparison of the rate coefficients shows that while transitions up to the pB_2_ intermediate are faster in the aqueous sample, those after the pB_2_ intermediate are up to an order of magnitude slower.

**Table 1:** Molecular rate coefficients obtained from the global spectrotemporal fit of the photocycle scheme (Fig. 3) to the present data (rehydrated) and to the 0.66 M NaCl, pH 8.2 data (Khoroshyy et al. 2013) (aqueous)

Transition	Rate coefficient (s^−1^)	
	Rehydrated	Aqueous
pR1 to pR2	$4.8\cdot10^{4}$	$3.7\cdot10^{5}$
Reverse	$2.7\cdot10^{2}$	$1.6\cdot10^{2}$
pR2 to pB1	$6.7\cdot10^{2}$	$2.2\cdot10^{3}$
Reverse	$1.0\cdot10^{3}$	$1.8\cdot10^{3}$
pB1 to pB2	$7.8\cdot10^{2}$	$1.0\cdot10^{3}$
Reverse	$1.6\cdot10^{2}$	$2.2\cdot10^{2}$
pB2 to pG1	16	2.9
Reverse	7.9	0.19
pG1 to pG	2.9	0.21

### Light-induced refractive index change of the photoactive yellow protein

With the pulsed excitation, the refractive index change of the sample was determined by monitoring the outcoupled light intensity at an incident angle of the measuring beam (HeNe, *λ* = 632.8 nm) tuned slightly off-resonance, to the half-maximum of the peak intensity. Measurements were taken on both sides of the resonance peak, and the absolute values were averaged. The experiments were performed at low- (20%) and high- (75%, 85%) relative humidity environments. For each different environment, the change in refractive index was monitored at two timescales (50 ms and 2 s full scales) after excitation. An additional measurement was taken on the sample at 85% relative humidity and 500 µs time scale, for the comparison of the OMA and OWLS results regarding the fast transition in the first part of the photocycle.

At low humidity (20%, data not shown) and 50 ms after excitation, the decay of the signal (guided light intensity change after the excitation of the PYP layer) was still in progress, and the calculated relative refractive index change was ∼ 10^−5^, which is comparable to the accuracy of our OWLS set-up. By 2 s after excitation, the measured intensity change was negligible. Comparing the OWLS results to the measured kinetics of the PYP, we concluded that the experienced refractive index change at low humidities stems from the heat jump induced by the exciting light, rather than the protein entering the photocycle. This observation is in agreement with the results of van der Horst et al. (2005).

**Fig. 5:**
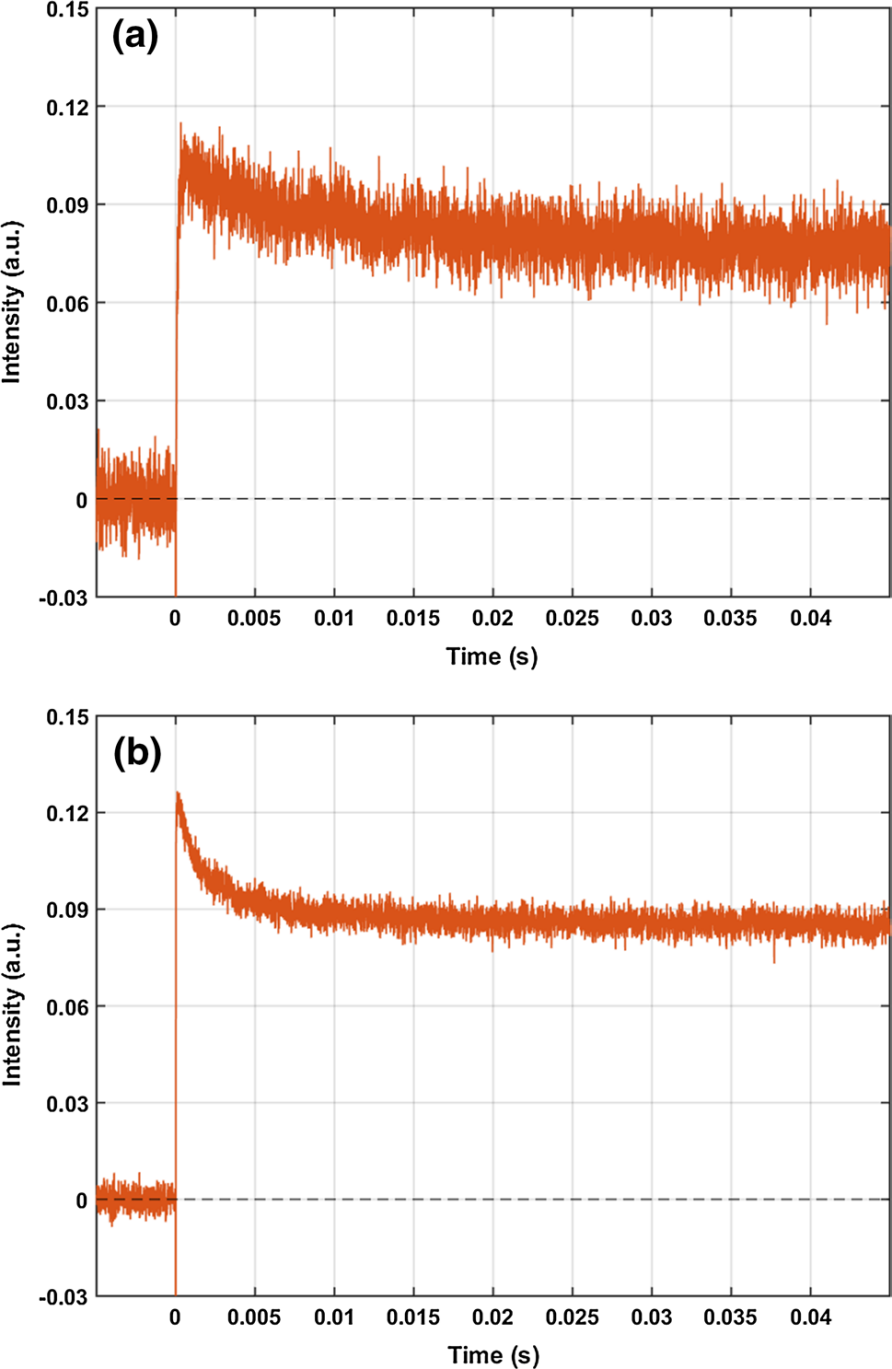
The measured OWLS data at 75% RH (**a**) and 85% RH (**b**). The data indicated are measured at the right side of the incoupling peak

**Fig. 6:**
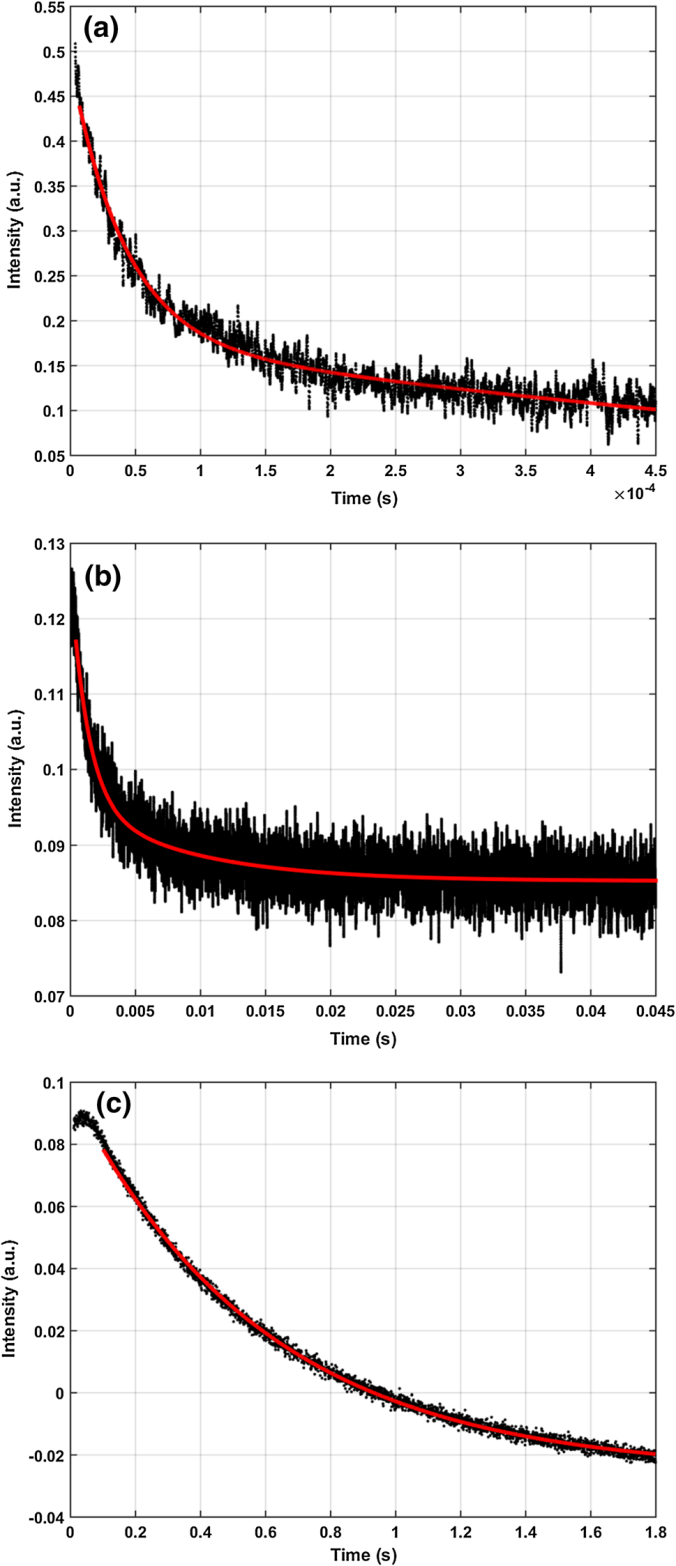
Exponential fit on the data measured in 85% RH environment on a 500-µs (**a**), 50-ms (**b**) and 2-second (**c**) timescale

At high humidities (both at 75% and 85%, Fig. 5), after 50 ms from the excitation a quasi-steady state is formed, whose refractive index is different from that of the ground state. The measured kinetics and concentrations at 85% relative humidity (shown in Fig. 4b) imply that at 50 ms after the excitation the mixture of two pB states dominates the photocycle, while the pR and pG_1_ conformations are present only in lesser amounts. The calculated refractive index change for the pB mixture state is $\Delta n = - 3\cdot 1 0^{ - 4}$. At 2 s after excitation, a small residual refractive index signal was present (Fig. 6c), which is likely due to a temperature artefact. At lower, 75% RH, the results were similar to those obtained at 85% RH.

At 85% relative humidity, an additional experiment was done on the 500-µs timescale, to compare the kinetic coefficients obtained from the OWLS and OMA experiments. The 500-µs timescale OWLS data were fitted with two exponentials, the second of them corresponding to the second phenomenological rate constant from the OMA results (Fig. 6a), while the first rate constant was found slightly slower than its absorption kinetic counterpart ($2.4 \cdot 10^{4} \,{\text{s}}^{ - 1}$). For the traces of 50-ms timescale, two exponentials were used, whose kinetic constants corresponded to the second and third phenomenological rate coefficients ($7.0 \cdot 1 0^{ 2}$, $1.44 \cdot 10^{2} \,{\text{s}}^{ - 1}$, respectively, Fig. 6b), while the 2-s timescale data could be fitted with one exponential with a rate constant of $1.7\;{\text{s}}^{ - 1}$ (Fig. 6c), which corresponds to the last phenomenological rate coefficient obtained from the OMA’s multiexpoential fit. A global multiexponential fit to the OWLS and OMA data on the 500-µs, 50-ms and 2-s timescales showed a similar correlation between the kinetics of the curves measured by the two methods, with a goodness of fit of *R*^2^ 0.96, 0.83 and 0.99, respectively, for the three timescales. These results confirm that the refractive index changes of the films are due to conformational changes in the protein molecule following light excitation.

At 85% relative humidity, an additional experiment with a blue-light CW excitation was also performed, where a refractive index change $\Delta n_{{{\text{pB}}-{\text{pG}}}} = - 5 \cdot 10^{ - 4}$ was observed at 632.8 nm (data not shown), and was attributed to a dynamic equilibrium between the pG and pB states. This value is slightly higher than the corresponding refractive index change observed during the photocycle ($- 3\cdot10^{ - 4}$), which might be explained by the < 1 quantum efficiency of the photocycle for a short flash, similarly to the BR-M transition in bacteriorhodopsin, where stationary excitation of the film yielded higher concentrations of the M form (Dér et al. 2007). Based on the amplitude ratios of the kinetic OWLS traces (Fig. 6a, b), the transient refractive index changes corresponding to the pR_1_ and pR_2_ states are estimated to be even higher (about 10 and 5 times, respectively), which are comparable to the maximum values obtained for bR films.

## Conclusions

The photocycle of dried PYP films was examined at different relative humidities. For determining the kinetics of the photocycle, we used an optical multichannel analyser set-up and compared the data to those of light-induced relative refractive index change measurements by optical waveguide lightmode spectroscopy.

At low humidity (20%), no regular photocycle takes place in the protein, and the small spectral changes observed are probably due to a temperature jump effect caused by the pumping laser.

At high humidities (75% and 85% RH), the photocycle is similar to the one measured in solution, but the rate-limiting steps are faster. In previous publications, a number of different photocycle schemes have been proposed for PYP in solution. According to our analysis, the simple, unbranched photocycle scheme containing multiple red- and blue-shifted intermediates (pR_1_, pR_2_, pB_1_, pB_2_ and pG_1_) with reversible transitions between most of the states, as established by (Khoroshyy et al. 2013), could adequately account for the photocycle of PYP in dried film at high RH (85%). With the time resolution of the experiments, the formation of pR_2_ from the pR_1_ state is the fastest transition with a phenomenological rate constant greater than $4.8 \cdot 10^{4} \,{\text{s}}^{ - 1}$. The spectra of the two pR states are similar to each other; however, they have sharper maxima than their counterparts in solution. The decay of pR and the formation of pB states are slower in the dried protein, with the pB states being less blue-shifted, and pB_1_ having spectral features resembling a mixture of, presumably, protonated and deprotonated chromophores. The refractive index change of blue-shifted intermediates is calculated to be $\Delta n = - 3\cdot10^{ - 4}$, while the faster phases of the OWLS signal indicate refractive index changes in the 10^−3^ regime, similar to the highest values of bR-based films.

The above results are expected to serve with essential information for future biophotonic devices utilizing the nonlinear optical properties of PYP or other chromoproteins. Note that optical film preparation at controlled pH and relative humidity is solved for chromoproteins like bR (see examples in Dér and Keszthelyi 2001 and references therein). According to our experience, optical-quality films of PYP, prepared at pre-adjusted pH, and stored under controlled relative humidity conditions, keep their functionality for at least 6 months. PYP could substitute or complement bR in such applications where, for example, shorter-wavelength operations are needed. Another distinct feature of PYP is that, contrary to bR, it is inherently water soluble in its monomeric form that allows its penetration into nanometric holes of porous silicon or other porous materials, making unique hybrid nonlinear optical structures possible. Most of all, applications in holography or integrated photonics are envisaged.
